# D-Serine Can Modify the Wall Teichoic Acid of MRSA via the *dlt* Pathway

**DOI:** 10.3390/ijms26094110

**Published:** 2025-04-25

**Authors:** Lei Wang, Jinru Xie, Qing Wang, Penghe Wang, Xinxin Hu, Tongying Nie, Lei Hou, Xinyi Yang, Xiukun Wang, Xuefu You, Congran Li

**Affiliations:** 1 Beijing Key Laboratory of Technology and Application for Anti-Infective New Drugs Research and Development, Institute of Medicinal Biotechnology, Chinese Academy of Medical Sciences & Peking Union Medical College, Beijing 100050, China; wanglei02250225@163.com (L.W.); xiejinru@163.com (J.X.); sywywph@foxmail.com (P.W.); xinxinhu@imb.cams.cn (X.H.); tongyingnie@imb.pumc.edu.cn (T.N.); xinyiyang@imb.cams.cn (X.Y.); xuefuyou@imb.pumc.edu.cn (X.Y.); 2 Division for Medicinal Microorganisms Related Strains, CAMS Collection Center of Pathogenic Microorganisms, Beijing 100050, China; 3 Key Laboratory of Major Diseases in Children, Ministry of Education, National Clinical Research Center for Respiratory Diseases, National Key Discipline of Pediatrics, Laboratory of Infection and Microbiology, Beijing Pediatric Research Institute, Beijing Children’s Hospital, Capital Medical University, National Center for Children’s Health, Beijing 100045, China; qingwang@bch.com.cn; 4 Experimental Animal Center, Institute of Medicinal Biotechnology, Chinese Academy of Medical Sciences & Peking Union Medical College, Beijing 100050, China; houlei0303@163.com; 5 State Key Laboratory of Bioactive Substances and Functions of Natural Medicines, Institute of Medicinal Biotechnology, Chinese Academy of Medical Sciences & Peking Union Medical College, Beijing 100050, China

**Keywords:** methicillin-resistant *Staphylococcus aureus* (MRSA), teichoic acid, enzyme kinetics, D-Serine, antibiotic resistance, sensitization

## Abstract

Methicillin-resistant *Staphylococcus aureus* (MRSA) infection is a serious clinical threat, and D-Serine (D-Ser) showed significant sensitization effects on β-lactams against MRSA in our previous study. Quantitative PCR analysis found the elevated expression of the *dlt* operon with D-Ser combination, which is responsible for wall teichoic acid (WTA) modification involving D-Alanine (D-Ala). This study aims to verify the effect of D-Ser on WTA modification through the *dlt* pathway and explore the related effects on bacteria. The DltA and DltC were recombined, and enzyme kinetic evaluations with different D-amino acids were then conducted; it was found that D-Ser is the second-best substrate for DltA (just after D-Ala), no matter whether DltC is present or not. D-Ser treatment also lowered WTA generation as demonstrated by WTA phosphate quantification and native-PAGE electrophoresis, increased the susceptibility of *S. aureus* to polymyxins, and elevated the mouse survival rate in the MRSA intraperitoneal infection model without affecting the bacterial loads in the main organs, indicating possible effects of D-Ser on MRSA virulence through WTA modification. In conclusion, the current study provided evidence for D-Ser modification of WTA via the *dlt* pathway, and its possible involvement in D-Ser sensitization deserves further investigation.

## 1. Introduction

The Gram-positive opportunistic bacteria *Staphylococcus aureus* widely exists in human living environments, and about 30% of healthy individuals carry *S. aureus* in their anterior nares [1]. *S. aureus* causes a variety of infections, from mild skin and soft tissue infections to more serious conditions such as endocarditis, pneumonia, and sepsis [1,2]. In particular, the emergence of methicillin-resistant *S. aureus* (MRSA) has posed a great challenge to the treatment and prevention of *S. aureus*.

D-amino acids are fundamental to microbial physiology. Different bacterial species produce and release various D-amino acids into the environment in millimolar range concentrations [3]. D-amino acids not only control the peptidoglycan chemistry, density, and strength of bacteria [4], but also regulate the biofilm dispersion and adhesion of certain bacteria [5]. Recent studies have revealed that D-Serine (D-Ser) inhibits the attachment and biofilm formation of MRSA [6].

Teichoic acid is a special component of the Gram-positive bacteria cell wall, including wall teichoic acids (WTA) attached to the peptidoglycan and lipoteichoic acids (LTA) linked to a membrane lipid [7]. WTAs are linear structures composed of glycerol-phosphate (GroP) or ribitol-phosphate (RboP) subunits polymerized by phosphodiester bonds [8]. These linear backbones of WTAs can be additionally modified by a variety of glycans and D-Alanine (D-Ala) [9]. WTAs are closely related to β-lactam sensitization [10], immune recognition [11,12], and virulence of bacteria [13,14], suggesting the great potential to become the next antibacterial active target.

D-Ala incorporation into WTAs requires four proteins encoded by the *dltABCD* operon [15]. *dlt* mutations caused changes in bacterial sensitivity in different Gram-positive bacteria. The absence of D-Ala esters from teichoic acids in *S. xylosus* and *S. aureus* leads to increased sensitivity toward cationic antimicrobial peptides (CAMP) [16]. The *dltA* mutant of *E. faecalis* is sensitive to the combination of cefotaxime (1 μg/mL) and amoxicillin (0.03 μg/mL) [17]. The Δ*dltABCD* mutant showed increased sensibility to bacitracin and vancomycin in *C. difficile* [18]. Perhaps, the *dltABCD* operon can be an effective target against Gram-positive bacteria.

Our previous research has shown that the combination of D-Ser can significantly increase the susceptibility of MRSA to β-lactam agents (oxacillin MIC from 16 μg/mL to 0.125 μg/mL and meropenem MIC from 8 μg/mL to 0.125 μg/mL) [19], and D-Ser had synergistic effects in combination with β-lactams against MRSA strains both in vitro and in vivo. In the mechanism study of D-Ser’s sensitization effect on β-lactams against MRSA, we found the elevated expression of the *dltABCD* operon after D-Ser treatment. The current study aims to explore the related mechanisms, and hence to provide information for understanding D-Ser’s sensitization effect.

## 2. Results

### 2.1. RNA Sequencing (RNA-Seq) Analysis

To explore the mechanism of D-Ser sensitization, we compared the expressions of different genes in D-Ser versus control groups, and meropenem (MEM) and D-Ser versus MEM groups. The differential gene expression (DEG) was screened using |log_2_(FoldChange)| ≥ 1 and *p*-value ≤ 0.05 as the cutoffs. In the differential expression of genes and the subsequent gene enrichment analysis of MEM and D-Ser versus MEM groups—the *dltABCD* operon involving in teichoic acid biosynthesis—D-amino acids metabolism, CAMP resistance, *S. aureus* infection, etc., caught our attention (Table S1 and Table 1).

Further analysis of the *dlt* operon expressions found significant changes with the combination of D-Ser (Table 2). The expression of *dlt* operon genes decreased after MEM treatment of MRSA N315. However, with the combination of D-Ser, the expression of the operon was up-regulated about two times and returned to the level of approximately the control group. D-Ser itself also elevated the expression of the *dlt* operon in MRSA N315, even though the extent was lower than in combination with MEM.

### 2.2. Quantitative Real-Time PCR (RT-qPCR) Result

**Figure 1:**
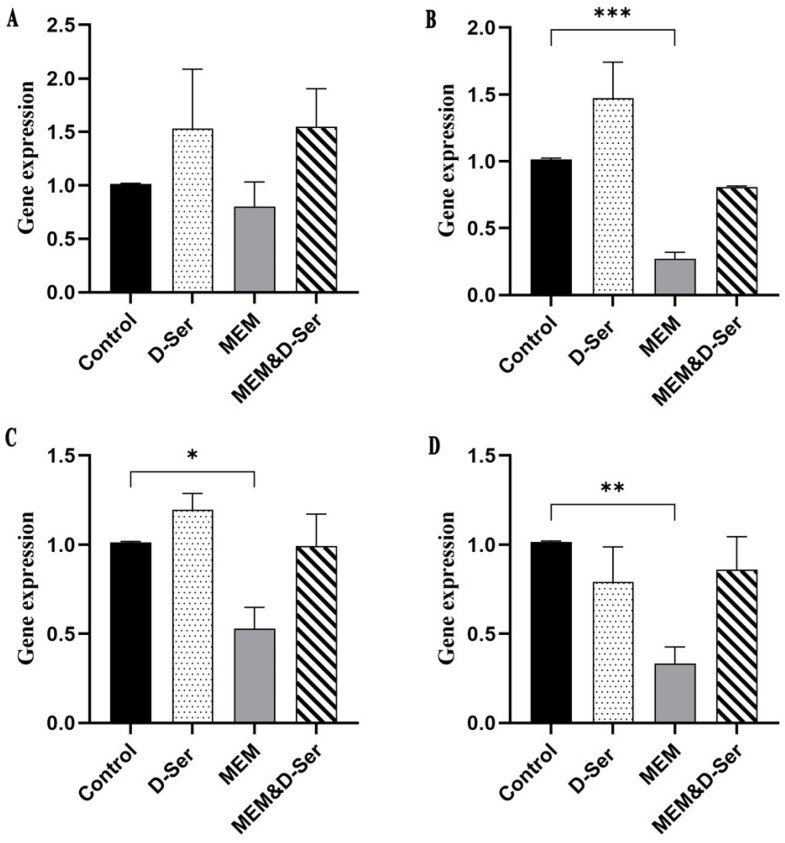
RT-qPCR quantification of the expressions of the *dlt* operon genes. (**A**) *dltA*, (**B**) *dltB*, (**C**) *dltC*, (**D**). *dltD*. Error bars represent the SDs from three biological replicates. Statistical significance was evaluated by the *t*-test. * *p* < 0.05; ** *p* < 0.01; *** *p* < 0.001.

RT-qPCR further confirmed the RNA-seq results (Figure 1). The results of RT-qPCR showed that the expression of *dltABCD* of the MEM group was significantly lower than that of the control group with the exception of *dltA* (about 0.80, 0.27, 0.53 and 0.33 folds for *dltA*, *dltB*, *dltC* and *dltD*, respectively). After combination with D-Ser, the expression level of *dltABCD* was about two-fold higher than that of the MEM group, and the levels were higher (*dltA*, 1.55-fold) or similar to (*dltBCD*, 0.81 to 0.99 folds) those of the control group.

### 2.3. S. aureus DltA Protein Can Use D-Ser as a Substrate

To investigate whether SaN315 DltA can use D-Ser as well as other D-amino acids as substrates, we overexpressed SaN315 DltA and measured the initial rates of the adenylation reaction catalyzed by SaN315 DltA via pyrophosphate-detection assays in the presence of various concentrations of different D-amino acids. An initial screening demonstrated that D-Ala, D-Ser, D-Threonine, and D-Proline had measurable activity, while all other D-amino acids did not show obvious activity in the highest soluble concentrations. Therefore, these four D-amino acids were selected as substrates to determine the enzyme kinetic parameters of DltA.

**Figure 2:**
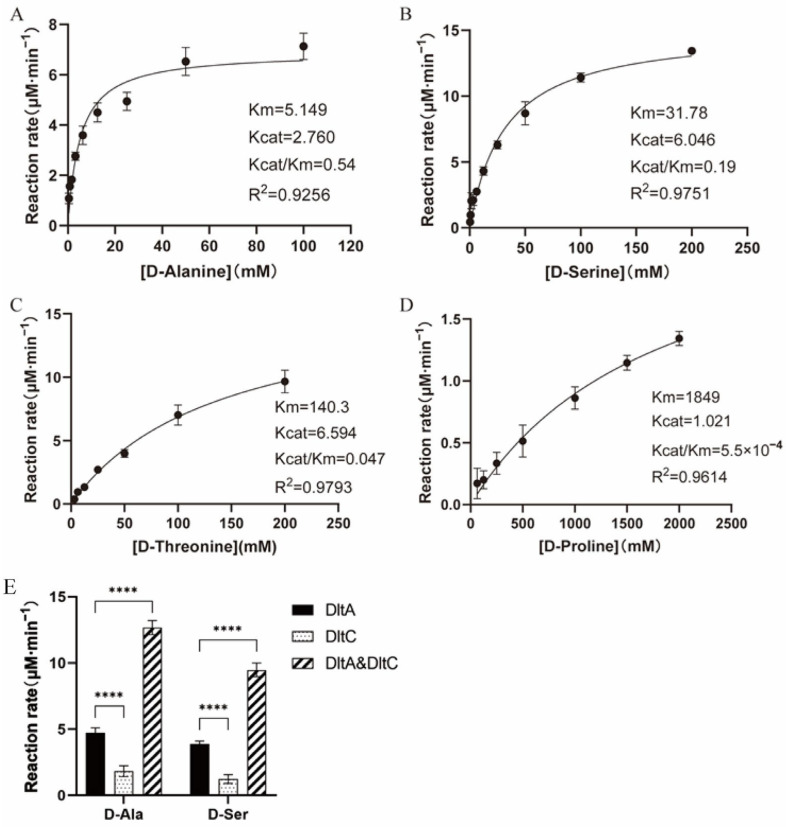
The adenylation reaction catalyzed by *S. aureus* N315 (SaN315) DltA with different D-amino acids as the substrates. (**A**–**D**). Michaelis–Menten fitting of the adenylation reaction catalyzed by SaN315 DltA with D-Ala, D-Ser, D-threonine, and D-proline as the substrates, respectively. The values from three to four measurements were used. The units for Km, Kcat and Kcat/Km are mM, min^−1^ and mM^−1^·min^−1^, respectively. (**E**). Effects of DltC on the reaction rate of DltA with D-Ala or D-Ser as the substrate. Initial rates of adenylation catalyzed by SaN315 DltA (2.5 μM) with D-Ser (2.5 mM) or D-Ala (2.5 mM) as the substrate, in the absence or presence of SaN315 DltC (125 μM), were measured. Error bars represent the standard deviation (*n* = 4). Statistical significance was evaluated by a two-way ANOVA multiple comparison test. **** *p*< 0.0001.

The results are shown in Figure 2A–D. D-Ala, the optimal substrate of N315 DltA, showed the lowest *K*_m_ value (5.149 mM) and the highest catalytic efficiency (*k_cat_*/*K*_m_ of 0.54 mM^−1^·min^−1^). D-Ser was the second-best substrate of N315 DltA, with *K*_m_ of 31.78 mM (about six-fold higher than that of D-Ala) and the *k_cat_*/*K*_m_ of 0.19 mM^−1^·min^−1^ (about three-fold lower than that of D-Ala). D-Threonine and D-Proline were relatively poor substrates for N315 DltA, with *K*_m_ values about 27-fold and 360-fold higher, and the *k_cat_*/*K*_m_ values around 11-fold and 980-fold lower. These results suggest that in addition to D-Ala, D-Ser can be used efficiently as a substrate for N315 DltA. We determined the MIC of MEM against MRSA strains by combining it with each of the above three D-amino acids (Table S2). The results showed that only D-Ser exhibited sensitizing activity.

To verify the effect of DltC on DltA reaction rate against D-amino acids, we overexpressed SaN315 DltC and further measured the initial rates of the adenylation reaction catalyzed by SaN315 DltA with D-Ala or D-Ser as the substrate in the presence or absence of SaN315 DltC (Figure 2E). DltC can also react with D-Ala and D-Ser, but the reaction efficiency was very low. When enough DltC was added to the DltA reaction system, the reaction efficiency of D-Ala and D-Ser was greatly improved (from 4.73 μM·min^−1^ to 12.67 μM·min^−1^ for D-Ala, and 3.88 μM·min^−1^ to 9.49 μM·min^−1^ for D-Ser). However, the reaction rate of D-Ser was still slightly lower than that of D-Ala (9.49 μM·min^−1^ versus 12.67 μM·min^−1^).

### 2.4. D-Ser Can Sensitize E. faecalis to MEM with Effects on DltA

**Figure 3:**
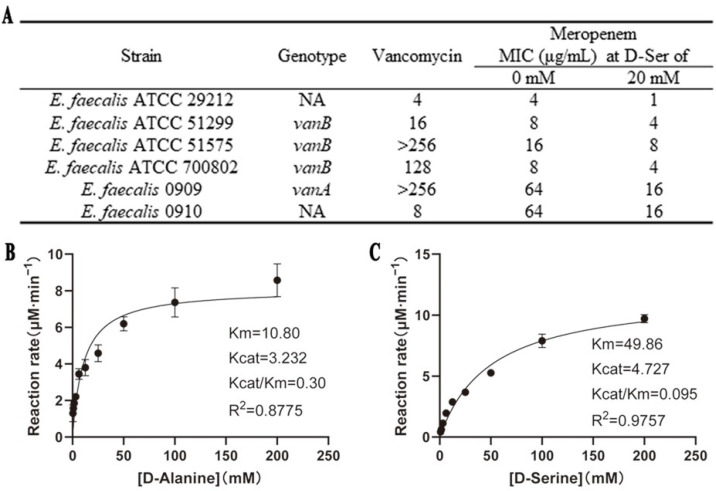
Effects of D-Ser on the antibacterial activity of MEM and DltA in *E. faecalis*. (**A**). MICs of MEM in combination with 20 mM D-Ser against *E. faecalis*; (**B**,**C**). Michaelis–Menten fitting of the adenylation reaction catalyzed by *E. faecalis* 29,212 DltA with D-Ala or D-Ser as the substrate. The values from three to four measurements were used. The units for Km, Kcat and Kcat/Km are mM, min^−1^ and mM^−1^·min^−1^, respectively.

In our previous research, the combination of 20 mM D-Ser can significantly increase the susceptibility of *S. aureus* to MEM [19]. We wonder if a similar effect exists in other Gram-positive bacteria like *E. faecalis*. The results in Figure 3A show that 20 mM D-Ser could decrease the MICs of MEM against *E. faecalis* by two to four folds, but the sensitizing effect was weaker than that in *S. aureus*.

Because *E. faecalis* and *S. aureus* have similar WTA structures [20,21], we blasted the gene and protein sequence of *S. aureus* and *E. faecalis* from the NCBI website (Table S3). A total of 33% of the *dlt* operon sequences of *E. faecalis* were matched with those of *S. aureus*, and there was 69.19% similarity. The *dltA dltB* and *dltC* had similarities of 64.72%, 69.19%, and 67.54%, respectively. Meanwhile, the amino acid sequence similarities of DltA to DltD were 30.00%~45.57%. We then overexpressed the *E. faecalis* 29,212 DltA by a similar method, as SaN315 DltA, and measured the initial rates via pyrophosphate-detection assays (Figure 3B,C). Compared to D-Ala, the *K*_m_ of D-Ser (49.86 mM) was about five-fold higher, and the *k_cat_*/*K*_m_ (0.095 mM^−1^·min^−1^) was about three-fold lower.

### 2.5. The Combination of D-Ser Can Reduce WTA Synthesis

**Figure 4:**
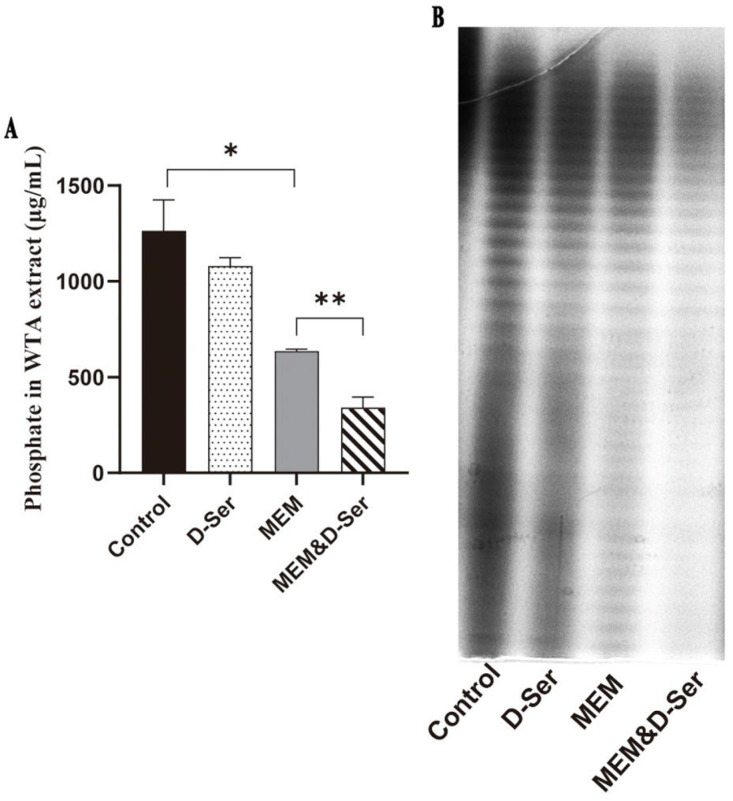
Effects of D-Ser on WTA contents and PAGE band patterns. (**A**). Phosphate contents of WTA extracts from MRSA N315 strain after different treatments. (**B**). Native-PAGE analysis of MRSA N315 WTA extracts after different treatments. * *p* < 0.05; ** *p* < 0.01.

Although the above experiments demonstrate that D-Ser can participate in the modification of the WTA, there is no direct evidence that D-Ser affects the WTA. Therefore, WTAs from MRSA N315 cultured with or without different drugs were extracted for comparison. Figure 4A shows that MEM can significantly reduce WTA content (1264.3 versus 635.3 μg/mL for the control and MEM groups, respectively, *p* < 0.05). After combination with D-Ser, WTA content decreased further (340.6 μg/mL for the MEM and D-Ser group), and there was a significant difference compared with the MEM group (*p* < 0.01). At the same time, native-PAGE analysis was used to visualize WTA band patterns and evaluate the relative abundance of WTAs of different lengths, because WTA-PAGE unique “laddering” of bands is generated by WTA chains of different lengths [22,23]. In Figure 4B, the WTA band patterns did not change significantly, but the WTA abundances were significantly reduced in the MEM and D-Ser group.

### 2.6. In-Vitro Activity of D-Ser in Combination with Cationic Antimicrobial Peptides Against S. aureus

According to the enrichment results of the KEGG pathway, the *dlt* operon is correlated with CAMP resistance. To verify this effect, we determined the MICs of D-Ser combined with polypeptide antibiotics against *S. aureus*. As shown in Table 3, *S. aureus* was susceptible to both vancomycin and daptomycin, and the D-Ser combination had no apparent effects. However, the addition of D-Ser can reduce the MICs of colistin two to four folds, and polymyxin B two to eight folds, although the resulting MICs were still higher than the corresponding breakpoints against *S. aureus*.

### 2.7. The Effect of D-Ser on the Surface Charge of MRSA N315

**Figure 5:**
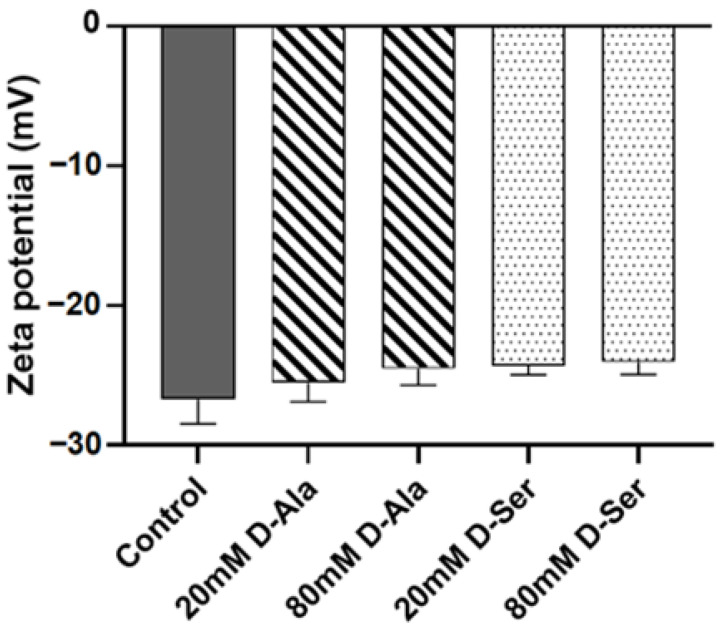
Effect of D-amino acids on the surface charge of MRSA N315. Data are expressed as mean ± SD (*n* = 3). Differences between groups were analyzed using *t*-test, and there were no significant differences.

To check the effects of D-Ser on the bacterial surface charges, we conducted zeta potential measurements. The results (Figure 5) showed that similar to D-Ala (zeta potentials of −25.53 mV and −24.53 mV for D-Ala 20 mM and 80 mM, respectively), there were no significant effects on bacterial surface charges with D-Ser (zeta potentials of −24.30 mV and −24.03 mV for D-Ser 20 mM and 80 mM, respectively) treatments in comparison to the control (zeta potential of −26.70 mV), consistent with the similar chargeability of the two D-amino acids.

### 2.8. D-Ser Can Reduce the Virulence of MRSA N315 In Vivo

**Figure 6:**
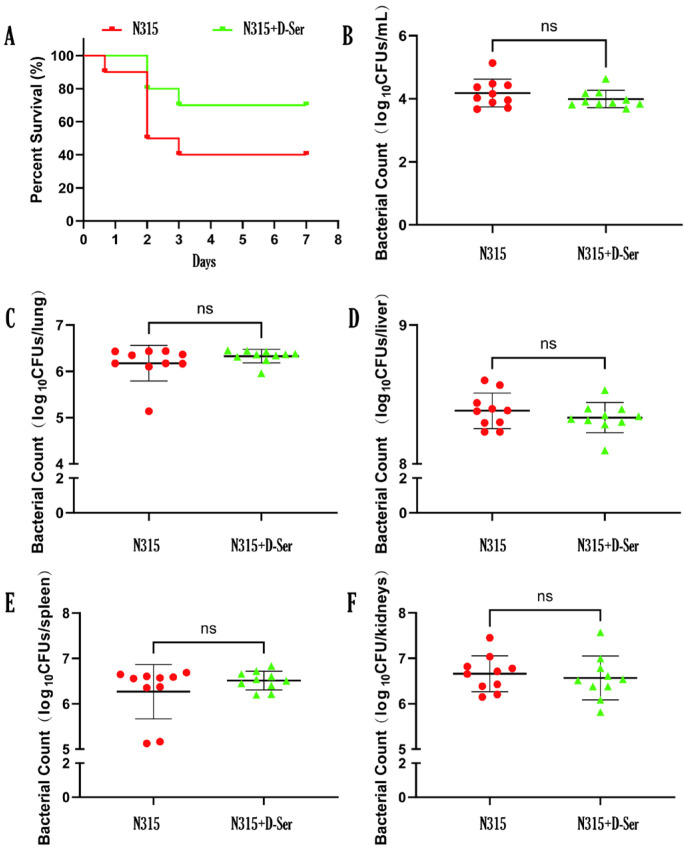
Effect of D-Ser addition on the virulence of MRSA N315 in mice. (**A**) The 7-day survival rate of each group; (**B**–**F**). Bacterial loads in blood and different organs of the mice after MRSA N315 infection. N315: the infection control group with intravenous infection of MRSA N315; N315 + D-Ser: the N315 + D-Ser group with intravenous injection of D-Ser 1 h after intravenous infection of MRSA N315.

The mice were infected with MRSA N315 via intravenous injection of 0.4 mL bacterial suspension properly diluted in saline; 1 h after infection, the mice were intravenous injected with 0.4 mL of 20 mM D-Ser (N315 + D-Ser group) or saline (N315 group). The results are shown in Figure 6A. For the N315 group, the mice began to die at 16 h after infection, and the survival rate of mice after 7 days was 40%. Intravenous injection of D-Ser 1 h after infection reduced the mortality, and the survival rate of mice increased to 70% after 7 days. To rule out the killing effect of D-Ser on bacteria, different organ and blood samples were collected for each group, and bacterial loads were counted. As shown in Figure 6B–F, there was no difference in bacterial loads between the two groups (4.18 versus 3.99 log_10_CFUs/mL for blood, 6.18 versus 6.33 log_10_CFUs/mL for lung, 8.38 versus 8.33 log_10_CFUs/mL for liver, 6.27 versus 6.51 log_10_CFUs/mL for spleen, and 6.66 versus 6.57 log_10_CFUs/mL for kidneys), suggesting that the lowered mortality rate in mice after the D-Ser addition was the result of the weakened virulence of strain by D-Ser, rather than the killing effect on bacteria. To expand the markers of infections in the mouse studies, we gauged the levels of inflammation and tissue damages by hematological analysis and biochemical analysis. The results (Figures S1 and S2) demonstrated that D-Ser administration did not cause significant differences on the indices checked in comparison to the N315 infection groups; however, D-Ser administration (N315 + D-Ser group) did alleviate some indices to certain extents in comparison to the N315 group at some time points, including hematological indices of ALY count, BAS count, LIC count, NEU count, PLT count, and biochemical indices of ALT, AST, Crea, Urea, LDH, α-HBHD, CRP, etc.

## 3. Discussion

Methicillin-resistant *Staphylococcus aureus* (MRSA) is considered to be a virulent epidemic pathogen, involving in bloodstream infections with high mortality rates [24]. MRSA with multi-drug resistance is becoming increasingly common in healthcare and community settings, and vancomycin has become the last line of defense against MRSA infections. Many studies are devoted to find new antimicrobial targets or small molecule drugs, natural products, or composite materials with synergistic sensitization activities [25,26,27,28].

Our previous study reported the significant sensitization effect of D-Ser on the anti-MRSA activity of β-lactam antibiotics both in vitro and in vivo [19], however, the specific mechanisms remain to be fully elucidated. To explore the mechanism, transcriptome analysis was performed, and elevated expression of the whole *dlt* operon was found in the MEM and D-Ser combination group in comparison to the MEM-only group, which was further confirmed by the RT-qPCR measurements. KEGG pathway analysis demonstrated that the *dlt* operon participates in teichoic acid biosynthesis, D-amino acids metabolism, CAMP resistance, *S. aureus* infection, etc., suggesting the possible involvement of *dlt* operon in D-Ser sensitization in MRSA.

We first studied the effect of D-Ser on teichoic acid biosynthesis by investigating if SaN315 DltA can use D-Ser as the substrate in addition to D-Ala, the natural modifier for WTA [29]. The results showed that D-Ser was the second-best substrate for SaN315 DltA just after D-Ala, while other D-amino acids with no sensitization effects can hardly be used as the substrates or with largely lowered activity. The DltA from *E. faecalis* was then overexpressed and used in the enzymatic kinetic study (Figure 3B,C), and the results demonstrated that the activity difference between D-Ala and D-Ser with *E. faecalis* DltA was extremely similar to that with *S. aureus* DltA. A comparison of the results with different DltA found lower activity of D-Ser with *E. faecalis* DltA than *S. aureus* DltA, consistent with the weaker sensitization effect of D-Ser on MEM against *E. faecalis* than *S. aureus*.

DltC is a kind of D-Ala carrier protein, which plays an important role in the translocation of D-Ala from cytoplasm to WTA. DltA transfers D-Ala to DltC in an ATP-dependent manner, and DltC presents D-Ala to DltB [30]. In addition, with the participation of DltD, D-Ala migrates across the membrane and onto teichoic acid [31,32]. The effect of DltC on the DltA reaction rate against D-Ala or D-Ser was then evaluated by the initial rate measurements of the adenylation reaction catalyzed by SaN315 DltA in the presence or absence of SaN315 DltC via pyrophosphate-detection assays (Figure 2E). The results showed that the reaction efficiency of D-Ala and D-Ser was greatly improved with the addition of enough DltC, even though the reaction rate of D-Ser was still slightly lower than that of D-Ala, indicating that DltA can deliver D-Ser to DltC, and D-Ser can replace D-Ala to participate in the *dlt* pathway.

The effect of D-Ser on WTA biosynthesis was then directly assessed by WTA extraction and quantifications (Figure 4). The results demonstrated that D-Ser alone could only slightly reduce WTA content in comparison to the no-treatment control. However, the WTA content was significantly decreased when D-Ser was combined with MEM. The N-acetyl glucosamine (GlcNAc) in the WTA skeleton is attached to the N-acetyl muramic acid (MurNAc) of peptidoglycan by a phosphodiester bond [15,33]. Hence, the destruction of peptidoglycan by MEM may further hinder WTA connectivity, which may result in a significantly lower level of WTA in the combined group.

Polymyxins are generally thought to have an antibacterial effect by displacing cations (Mg^2+^, Ca^2+^) from the outer membrane of Gram-negative bacteria, thereby increasing the permeability of the bacterial membrane; however, they do not affect Gram-positive bacteria [34]. However, there is emerging evidence that WTA may be a target for polymyxin B to kill Gram-positive bacteria, and its resistance is related to the electronegativity of the surface [35]. Previous reports indicated that the positive charge modification by D-Ala can neutralize the electronegativity of WTA [15,36], and changes in the electronegativity or structure of WTA may be the reason for sensitizing colistin and polymyxin B [35]. Hence, the replacement of D-Ala by D-Ser through the *dltABCD* pathway to modify WTA may play a role in the change of polymyxin MICs in combination with D-Ser. Besides, *dlt* operon-mediated D-alanylation is a key step in masking the negative charge of the cell wall. Hence, the replacement of D-Ala by D-Ser in WTA modification, which may cause charge or structure change of WTA, could affect the susceptibility of the Gram-positive bacteria to polymyxins. Additionally, the *S. aureus* Δ*dltA* mutant with less hydrophobicity was reported to be more susceptible than wild type *S. aureus* to a cationic antimicrobial polymer [37]. Consistent with the higher hydrophilicity of D-Ser in comparison to D-Ala, D-Ser reduced the MICs of polymyxin B and colistin against MRSA in our study. It is also reported that the increase in the positive charge of the cell wall is thought to contribute to the rejection of daptomycin [38,39]. The combination of D-Ser did not affect the MIC of daptomycin in the present study, consistent with the zeta potential results that D-Ser did not have much of an effect on bacterial surface charge (Figure 5).

Due to the changes in surface charge, D-Ala modifications are considered key determinants for the formation of *S. aureus* biofilms [40]. In the case of inactivation of *dltABCD* or defects in WTA, the loss of D-alanine residues increases the negative charge on the cell surface, limiting the formation of biofilms and the adhesion ability of bacteria [40]. However, D-Ser alone had no obvious effects on the formation and degradation of *S. aureus* biofilms in vitro in our preliminary experiments. Even though the results are consistent with our zeta potentiometric determination data that D-Ser had little effects on the bacterial surface charge of *S. aureus* (Figure 5), they may seem contradictory to previous reports [6]. More experiments using different in-vitro and in-vivo models are needed for further evaluations.

The *dlt* operon is essential for regulating the D-Ala modification of WTA, and WTA can promote the release of surface toxins [41]. Collins et al. demonstrated that *dlt* deficient *S. aureus* is less virulent in mice, which had significantly lower rates of sepsis and septic arthritis [24]. Moreover, WTA can mediate various reactions between *S. aureus* and the host, such as colonization, pathogenicity, and induction of the immune response [42]. In addition, in other Gram-positive bacteria, the *dlt* operon is also a key factor influencing virulence. The *dlt* operon can regulate cariogenic virulence in *Streptococcus mutans* [43], and the *dltA* mutation can affect the expression of virulence factors in *Streptococcus pyogenes* [44]. In the present study, as D-Ser can be incorporated into WTA synthesis via the *dlt* operon, we evaluated the effect of D-Ser on the in-vivo virulence of the bacteria by the mouse intravenous systemic infection model (Figure 6). The results demonstrated that with the administration of D-Ser, the survival rate of the mice increased by 30%, while the corresponding bacteria loads in major organs and blood did not demonstrate differences, suggesting a virulence change of the bacteria by D-Ser. The results may seem contradictory to our previous report that D-Ser alone has no therapeutic effect in mouse systemic infection by MRSA [19]. The inconsistency may be related to the different infection routes (intravenous versus intraperitoneal) and D-Ser administration routes (intravenous versus subcutaneous); hence, D-Ser had more chance to affect the virulence of the bacteria in the current study. In addition, the D-Ser’s effect on bacterial virulence can only be seen with specific infection doses, like the dose that caused the death of 60% of mice in the present study; the minimal lethal dose used in the previous study may be too high to demonstrate D-Ser’s effect on virulence. Consistent with these, the hematological analysis and biochemical analysis of the inflammation and tissue damages indices suggested that D-Ser administration did not cause significant effects on the indices checked, but alleviated some related indices at certain time points (Figures S1 and S2).

## 4. Materials and Methods

### 4.1. Bacterial Strains, Media, Vectors, and Growth Parameters

The methicillin-resistant *S. aureus* (MRSA) strain N315 (MRSA N315) was used as the wild-type strain. Tryptic soy broth (TSB) and Luria Bertani broth (LB) were used for *S. aureus* and *E. coli* growth, respectively. Unless otherwise specified, bacterial cultures were recovered in an agar plate from −80 °C and grown in TSB (*S. aureus*) or LB (*E. coli*) at 37 °C with shaking at 220 rpm. pCold I, a cold shock express vector for the recombinant expression of proteins, was obtained from ZOMANBIO (Beijing, China). For plasmid maintenance, growth media were supplemented with 50 μg/mL ampicillin. The primers, plasmids, recombinant vectors, and bacterial strains used in this study are listed in Table S4.

### 4.2. RNA Extraction and RNA Sequencing (RNA-Seq)

Four groups were set up in the experiment: MRSA N315 without drugs (control group), MRSA N315 with 20 mM D-Serine (D-Ser group), MRSA N315 with 4 μg/mL Meropenem (MEM group), and MRSA N315 with 4 μg/mL MEM and 20 mM D-Ser (MEM and D-Ser group). The overnight MRSA N315 culture was 1:100 diluted in 30 mL TSB, and incubated at 37 °C, 220 rpm until OD_600_ = 0.2, at which point the appropriate drugs were added, and incubated for an additional 2 h. The bacterial cells were collected by centrifugation at 4 °C, 5000× *g*, for 10 min, and 5 mg/mL lysostaphin was added and incubated at 37 °C for 1 h to lyse the bacterial cell wall. Bacterial RNA was extracted using the RNeasy Mini Kit (Qiagen, Hilden, Germany). The experiments were repeated three times on different days. The RNA-seq was commissioned by Novogene (Beijing, China). Since the differential expression analysis in RNA-seq involves independent statistical hypothesis testing on a large number of gene expression values, there is a problem of false positives. Therefore, Padj-value is introduced to correct the significant *p*-value. The Benjamini–Hochberg (BH) method is used to control the false positive rate. The Padj-value reflects the significance level in the case of multiple tests. Typically, Padj < 0.05 is considered as the significant criterion, indicating that the differential expression of this gene is statistically significant.

### 4.3. Quantitative Real-Time PCR (RT-qPCR)

The same set of RNA was used for qPCR as for RNA-seq. An amount of 2 μg RNA was reverse transcribed into cDNA using a FastKing RT kit (with gDNase) (Tiangen, Beijing, China). A SYBR Green Master Mix (ABI) was used to determine the relative expression levels of the target genes. The primers used are shown in Table S4. *rpoB* [45] was used as the internal control in qPCR and the relative expressions of the target genes were analyzed by the 2^−ΔΔCt^ method. All samples were repeated three times in parallel.

### 4.4. Antimicrobial Susceptibility Test

According to the Clinical Laboratory Standards Institute, the minimum inhibitory concentration (MIC) was determined by the broth microdilution method in cation-adjusted Mueller-Hinton (CAMH) broth. The final inoculum in each well was about 5 × 10^5^ CFU/mL. After incubating at 37 °C for 18–20 h (24 h for vancomycin), the results were recorded by the naked eyes.

### 4.5. Cloning, Expression, and Purification of DltA and DltC

The DltA and DltC proteins were overexpressed and purified according to a report by Lee et al. [29] with modifications. The *dltA* and *dltC* genes of MRSA N315 and the *dltA* gene of *E. faecalis* 29,212 were PCR amplified using primers listed in Table S4. The *dltA* PCR product of *S. aureus* was digested by *Xho* I (Takara, Japan) and *Hind* III (Takara, Japan) restriction enzymes, the *dltC* PCR product of *S. aureus* was digested by *Nde* I (Takara, Japan) and *Xba* I (Takara, Japan), and the *dltA* PCR product of *E. faecalis* 29,212 was digested by *Nde* I (Takara, Japan) and *Xho* I (Takara, Japan). All the restriction enzymes are incubated at 37 °C for 4 h. The digested PCR products were further incorporated into the pCold I expression vector (Zhuangmeng, Beijing, China) to construct the recombinant protein overexpression plasmids with an N-terminal His-6 tag.

The resultant plasmids were transformed into *E. coli* BL21(DE3) for overexpression of the target proteins. Briefly, *E. coli* BL21(DE3) carrying the recombinant expression plasmids were grown in an LB medium containing 50 μg/mL ampicillin at 37 °C, 220 rpm until OD600 ≈ 0.5; the overexpression of the proteins was induced by adding 0.5 mM IPTG and incubated at 15 °C, 220 rpm for 12 h. The bacterial cells were collected by centrifugation at 4 °C, 10,000× *g* for 5 min, resuspended in Buffer A (50 mM Tris-HCl, 500 mM NaCl, pH8.0), and disrupted by ultrasonication (3s on, 3s off, for 3 h) in ice water. The bacterial lysate was centrifuged at 4 °C, 12,000× *g* for 10 min, and the supernatant was collected and filtered by a 0.22 μm membrane. The filtered supernatant was then loaded on the Ni-NTA affinity column using the ÄKTA™ pure system (Cytiva, Marlborough, MA, USA) and eluted with imidazole gradience. The partially purified protein was concentrated to 500 μL by 30 kDa ultrafiltration tube, and further purified by Superdex 75 10/300 GL (Cytiva, Marlborough, MA, USA). The protein was again concentrated using a 30 kDa ultrafiltration tube and stored in Buffer C (20 mM Tris-HCl, 100 mM NaCl, pH 7.5). The purity of the protein was checked by 12% SDS-PAGE gel electrophoresis.

### 4.6. Enzyme Kinetics Assay

The activity of MRSA N315 and *E. faecalis* 29,212 DltA protein with different D-amino acid as the substrates was determined by a pyrophosphate assay (also known as malachite green ammonium phosphorus molybdate assay) [46,47,48] using Malachite Green Phosphate Detection Kit (Beyotime, Shanghai, China). The experimental reaction system was as follows: 50 mM Tris-HCl pH7.4, 100 mM KCl, 10 mM MgCl_2_, 5 U/mL inorganic pyrophosphatase (Beyotime, Shanghai, China), 2.5 μM DltA, 0.25 mM ATP, different concentrations of D-amino acids. Respectively, the concentration ranges of D-amino acids were as follows: 0.39 mM to 100 mM for D-Alanine (D-Ala), 0.39 mM to 200 mM for D-Ser, 3.125 mM to 200 mM for D-Threonine, and 62.5 mM to 2000 mM for D-Proline. The reaction was started by ATP addition, continued at 37 °C for 20 min, and stopped by adding 70 μL of the mixed reagent A and reagent B (at a ratio of 2:1) from Malachite Green Phosphate Detection Kit (Beyotime, Shanghai, China) and incubated at room temperature for 20 min. The absorbance at 630 nm was detected by the Enspire 2300 multi-mode microplate reader (PerkinElmer, Waltham, MA, USA). Each reaction system has three parallel repeats, and the reaction system without substrate was used as the blank control. Rate-concentration curves against D-amino acids were fitted to the Michaelis–Menten equation using GraphPad prism 9.5.1 to calculate *K*_m_ and *k_cat_*.

To determine the effect of DltC on the enzymatic reaction activity of DltA, the DltA reaction rate in the presence or absence of 125 μM DltC protein was measured, with D-Ser (2.5 mM) or D-Ala (2.5 mM) as the substrate [49].

### 4.7. WTA Extraction and Quantification

Wall teichoic acid (WTA) was extracted by methods reported by Kho et al. [23] with modifications. Overnight cultures of single colonies were 1:100 diluted in 40 mL fresh TSB and incubated at 37 °C, 220 rpm for 2 h. Different drugs were then added and cultured at 30 °C, 200 rpm for an additional 16~20 h until the stationary phase. The same amount of bacterial cells for different strains was collected by centrifugation at 7000× *g*, 4 °C for 15 min. The cell pellets were washed using Buffer 1 (50 mM MES, pH 6.5), resuspended in 20 mL Buffer 2 (50 mM MES, 4% (*w*/*v*) SDS, pH 6.5), and incubated in boiling water for 1 h. The suspensions were again centrifuged, and the pellets were washed with Buffer 2 three times, Buffer 3 (50 mM MES, 2% NaCl, pH 6.5) once, Buffer 1 once, and then resuspended in 2 mL Buffer 4 (20 mM Tris-HCl, 0.5% SDS, pH 8.0) containing 20 μg/mL proteinase K, and incubated at 50 °C for 4 h with 1000 rpm shaking. The pellets were collected by centrifugation at 16,000× *g* for 5 min, washed with 2 mL Buffer 3 three times, distilled H_2_O once, and then resuspended in 1 mL 0.1 M NaOH and incubated at 25 °C for 16 h with 1000 rpm shaking. After centrifugation (16,000× *g*, 15 min), the supernatants containing the crude WTAs were collected, and 250 μL of 1 M Tris-HCl, pH 7.8 were added to neutralize the solutions.

Since perchloric acid is a strong oxidizing agent, we used it to decompose the samples and oxidize the organophosphates in WTA to orthophosphates. Under acidic conditions, ammonium molybdate is reacted with phosphoric acid to form ammonium phosphomolybdate complex, which is reduced to the molybdenum blue complex by ascorbic acid. The determination of absorbance at 820 nm is the content of phosphorus in WTA. The total amounts of WTAs in different groups were quantified by determining the total amount of inorganic phosphate in the WTA extracts [50]. Different volumes (0, 40, 60, 80, 120, 160 μL) of 100.7 μg/mL KH_2_PO_4_ were used as a standard for the determination of standard curves. An amount of 50 μL perchloric acid was added to each lyophilized WTA extracted, and the resultant solution was transferred to glass test tubes. The test tube was gently heated over a flame, causing the released fumes to condense inside the tube wall. Then, 950 μL of water and 500 μL of P-reagent [2.2% (*w*/*v*) ascorbic acid solution, 0.33 M sulfuric acid solution, and 0.56% (*w*/*v*) ammonium molybdate solution] were added. After incubating at 37 °C for 90 min, the absorbance at 820 nm was recorded.

The WTAs extracted were separated by native PAGE (without SDS) with 30% [T], 2.6% [C] stacking gel, 20% [T], and 6% [C] separating gel, where [T] means total percentage concentration that refers to the total percentage concentration of the acrylamide and methylene bisacrylamide, and [C] means cross-linking percentage concentration that reflects the proportion of bisacrylamide in the total monomer. The samples were separated by electrophoresis at 4 °C under a constant current of 20 mA for about 20 h until the loading buffer dye reached the end of the gel, and was stained by alcian blue–silver.

### 4.8. Murine Systemic Infection Model

Four- to six-week-old CD-1 ICR mice were purchased from Spelford Biotechnology Co., Ltd. (Beijing, China), and acclimated to the environment for 3 days before the experiment. All animals were housed under controlled humidity (40 to 60%) and temperature (22 ± 2 °C) with a 12 h light-dark cycle. The animal study complied with the animal husbandry guidelines, with approval from the Laboratory Animal Welfare and Ethics Committee in Institute of Medicinal Biotechnology, Peking Union Medical College. The animals with behavioral abnormalities or injuries unrelated to the experimental protocol will be excluded, and for the current study, there were no exclusions of animals.

Three batches of animals were included in the study, for survival rate comparison, bacterial load comparison, and hematological and biochemical analysis, respectively. For the first two batches, the mice were randomized by weight into two groups (the N315 group and the N315 + D-Ser group, *n* = ten per group, five males and five females, the group size was chosen based on ethical and statistical requirements). MRSA N315 culture was freshly prepared and adjusted to proper density in 0.85%NaCl. After intravenous injections of 0.4 mL bacterial suspension (1.6~2.6 × 10^9^ CFU/mL), the N315 + D-Ser group was administered intravenously with 0.4 mL 20 mM D-Ser at 1 h after infection (saline for the control). For survival rate comparison, animal deaths were recorded for 7 days, the survival rates were calculated, and survival curves were plotted by GraphPad Prism 9.5.1 Kaplan-Meier survival curves analysis. For bacterial load comparisons, blood and organs (liver, spleen, lung, and kidneys) were collected after 12 h of infection and homogenized in saline, and the bacterial loads were counted by methods reported [19]. The results were reported in log_10_ values and the difference between the two groups was compared using *t*-test.

For the third batch, the mice were randomized by weight into 10 groups, the normal control group, the three N315 groups, the three N315 + D-Ser groups, and the three D-Ser groups (*n* = five per group, two males and three females, or three males and two females, the group size was chosen based on ethical and statistical requirements). MRSA N315 culture was freshly prepared and adjusted to proper density in 0.85%NaCl. The N315 groups and the N315 + D-Ser groups were intravenously injected with 0.4 mL bacterial suspension (2.1 × 10^9^ CFU/mL). Then, 1 h later, the D-Ser groups and the N315 + D-Ser groups were administered intravenously with 0.4 mL 20 mM D-Ser. The blood samples were harvested at 2, 4, and 12 h after infection (one group for each time point for the N315, N315 + D-Ser and D-Ser groups; blood samples for the normal control group were harvested at 2 h). The counts and percentage of atypical lymphocyte (ALY), basophils (BAS), eosinophils (EOS), large immature cell (LIC), lymphocytes (LYM), monocytes (MON), neutrophils (NEU), total white blood cells (WBC), and platelets (PLT) were measured using an ABX Micros 60 Hematology Analyzer (Horiba-ABX, Montpellier, France). The sera were obtained by centrifugation after the blood samples were left at 25 °C for 30 min. Serum levels of aminotransferase (ALT), aspartate aminotransferase (AST), total protein (TP), albumin (ALB), creatinine (Crea), urea, urine acid (UA), lactate dehydrogenase (LDH), α-hydroxybutyrate dehydrogenase (α-HBDH), and C-reactive protein (CRP) were measured using a Fully Automated Biochemistry Analyzer (Hitachi Diagnostic Products Corporation, Tokyo, Japan).

### 4.9. Zeta Potential Measurement

Single colonies of MRSA N315 were cultured overnight in TSB liquid medium at 37 °C and 220 rpm. The next day, the cultures were diluted 1:100 into fresh TSB liquid medium. Different concentrations of D-Ala and D-Ser were added, and the cultures were incubated at 37 °C and 220 rpm for 4 h. The bacterial cells were collected by centrifugation at 6000× *g* for 3 min, washed twice with sterilized ultrapure water, and finally resuspended in sterilized ultrapure water. The bacterial suspension was diluted 1:1000 in ultrapure water, and the zeta potential was measured using the Malvern particle size analyzer (Marven Nano ZS, Almelo, The Netherlands) three times. Zeta potentials were calculated from the electrophoretic mobility by the Henry equation at 25 °C, with three repeats per sample. The dispersant dielectric constant was set at 78.5. Viscosity was set at 0.8872 cP, and the dispersant refractive index was set at 1.330.

### 4.10. Statistical Analysis

Statistical analysis was performed by GraphPad Prism 9.5.1. P values were calculated using *t*-test or two-way ANOVA multiple comparison test to compare the differences between each pair of groups. A *p* value lower than 0.05 was considered statistically significant.

## 5. Conclusions

In summary, transcriptome analysis and RT-qPCR measurement suggested the involvement of a *dlt* operon (an operon participates in teichoic acid biosynthesis, D-amino acids metabolism, CAMP resistance, *S. aureus* infection, etc.) in D-Ser sensitization. The effects on teichoic acid biosynthesis were studied by an enzymatic kinetic study and WTA quantification. An enzymatic kinetic study using purified DltA from *S. aureus* as well as *E. faecalis* found that D-Ser was the second-best substrate for DltA, just after the natural substrate D-Ala. WTA quantification further confirmed the lower WTA contents with D-Ser treatment. MIC measurements found that D-Ser reduced the MICs of polymyxin B and colistin against MRSA, however, D-Ser did not demonstrate a significant effect on bacterial surface charge in zeta potential measurement. The in-vivo virulence analysis using a mouse intravenous systemic infection model demonstrated increased survival rate of the mice with D-Ser administration, while the corresponding bacteria loads in major organs and blood did not demonstrate a difference, suggesting virulence change of the bacteria by D-Ser. Hence, D-Ser involves in the *dlt* pathway through an ATP-dependent enzymatic reaction with a DltA protein, and functions in WTA modification, which may play a role in the D-Ser sensitization effect, and deserves further investigation.

## Figures and Tables

**Table 1:** The gene enrichments by KEGG pathway between MEM and D-Ser and MEM groups.

KEGG ID	Description	Padj	Gene name
sau01503	Cationic antimicrobial peptide (CAMP) resistance	0.02	*dltB/dltA/dltC/dltD*
sau00340	Histidine metabolism	0.026	*hutU/hutG/hutI/hisG*
sau02020	Two-component system	0.026	*lrgA/lrgB/uhpT/vraE/dltB/*SA_RS00475/*dltA/dltC/dltD*
sau02060	Phosphotransferase system (PTS)	0.026	SA_RS01835/SA_RS01830/SA_RS01840/SA_RS11260/SA_RS01110
sau00552	Teichoic acid biosynthesis	0.130	*dltB/dltA/dltC/dltD*
sau05150	*Staphylococcus aureus* infection	0.299	*sdrC/dltB/dltA/dltC/dltD*
sau00330	Arginine and proline metabolism	0.349	SA_RS08935/rocF
sau00470	D-Amino acid metabolism	0.497	*dltA/dltC*
sau00561	Glycerolipid metabolism	0.497	*dhaL/dhaK*

**Table 2:** Changes in gene expression of the *dlt* operon.

Gene Name	MEM vs. Control		D-Ser vs. Control		MEM & D-Ser vs. Control		MEM & D-Ser + vs. MEM	
	Log_2_FC	Padj	Log_2_FC	Padj	Log2FC	Padj	Log2FC	Padj
*dltA*	−0.541	<0.01	0.578	0.211	0.578	<0.05	1.129	<0.001
*dltB*	−0.798	<0.001	0.517	0.299	0.220	0.478	1.040	<0.001
*dltC*	−1.059	<0.001	0.480	0.469	0.227	0.546	1.301	<0.001
*dltD*	−1.093	<0.001	0.458	0.448	0.245	0.467	1.343	<0.001

When Padj < 0.05, we considered that there was a significant difference.

**Table 3:** MICs of polypeptide antibiotics against *S. aureus* in combination with 20 mM D-Ser.

Strain	MIC (μg/mL) of Polypeptide at D-Ser of							
	Vancomycin		Daptomycin		Colistin		Polymyxin B	
	0 mM	20 mM	0 mM	20 mM	0 mM	20 mM	0 mM	20 mM
MSSA ATCC 29213	0.5	0.5	1	0.5	512	256	128	64
MSSA12-02	1	1	1	1	1024	256	128	32
MRSA 08-50	1	0.5	1	1	512	128	128	32
MRSA N315	0.5	0.5	1	1	1024	256	128	64
MRSA ATCC 43300	1	1	1	1	>1024	256	256	32

## Data Availability

All data supporting the findings of this study are available in the paper and its Supporting Information or are available from the corresponding authors on reasonable request.

## Supplementary Materials

The following supporting information can be downloaded at: https://www.mdpi.com/article/10.3390/ijms26094110/s1.

## Abbreviations

The following abbreviations are used in this manuscript:

CAMP	Cationic antimicrobial peptide
D-Ser	D-Serine
D-Ala	D-Alanine
LTA	Lipoteichoic acids
MEM	Meropenem
MRSA	Methicillin-resistant *S. aureus*
WTA	Wall teichoic acid
