# Supplementary material for: D-Serine Can Modify the Wall Teichoic Acid of MRSA via the dlt Pathway
Source: Int J Mol Sci. 2025 Apr 25;26(9):4110. doi: 10.3390/ijms26094110 (PMC12071552; doi:10.3390/ijms26094110)
Supplement: Supplementary file 1 [file ijms-26-04110-s001.zip › ijms-3523366-supplementary.pdf]

Supplementary Information

Table S1. The expressions of differential genes between MEM & D-Ser and MEM groups

Gene name	Gene description	Log_2FC	*P*adj
SA_RS01835	PTS sugar transporter subunit IIB	-3.417	<0.0001
SA_RS01830	PTS ascorbate transporter subunit IIC	-2.972	<0.0001
SA_RS01840	PTS sugar transporter subunit IIA	-2.813	<0.0001
lrgA	antiholin-like murein hydrolase modulator LrgA	-2.392	<0.01
SA_RS01845	BglG family transcription antiterminator	-2.236	<0.0001
SA_RS01325	class I adenylate-forming enzyme family protein	-2.128	<0.01
SA_RS01230	sugar ABC transporter permease	-2.044	<0.0001
lrgB	antiholin-like protein LrgB	-1.986	<0.05
SA_RS01235	Gfo/Idh/MocA family oxidoreductase	-1.960	<0.0001
raiA	ribosome-associated translation inhibitor RaiA	-1.871	<0.0001
SA_RS01225	sugar ABC transporter permease	-1.818	<0.0001
SA_RS08625	universal stress protein	-1.766	<0.0001
butA	(S)-acetoin forming diacetyl reductase	-1.760	<0.0001
mltA	PTS sugar transporter subunit IIA	-1.759	<0.0001
SA_RS13725	hypothetical protein	-1.641	<0.01
hutU	urocanate hydratase	-1.587	<0.0001
SA_RS11255	BglG family transcription antiterminator	-1.514	<0.0001
dhaL	dihydroxyacetone kinase subunit DhaL	-1.484	<0.0001
mltD	mannitol-1-phosphate 5-dehydrogenase	-1.447	<0.0001
SA_RS01240	Gfo/Idh/MocA family oxidoreductase	-1.446	<0.001
gntP	gluconate:H+ symporter	-1.429	<0.0001
SA_RS04285	hypothetical protein	-1.426	<0.0001
uhpT	hexose-6-phosphate:phosphate antiporter	-1.387	<0.0001
SA_RS13540	DUF2316 family protein	-1.350	<0.05
SA_RS08935	proline dehydrogenase	-1.321	<0.0001
vraH	peptide resistance ABC transporter activity modulator VraH	-1.318	<0.01
rocF	arginase	-1.306	<0.0001
SA_RS01330	acyl CoA:acetate/3-ketoacid CoA transferase	-1.284	<0.0001
SA_RS06000	TM2 domain-containing protein	-1.248	<0.0001
vraE	peptide resistance ABC transporter permease subunit VraE	-1.241	<0.0001
SA_RS10150	phage tail family protein	-1.220	<0.001
SA_RS09985	hypothetical protein	-1.208	<0.0001
SA_RS10105	CHAP domain-containing protein	-1.191	<0.05
dhaK	dihydroxyacetone kinase subunit DhaK	-1.188	<0.0001
adaB	methylated-DNA--[protein]-cysteine S-methyltransferase	-1.186	<0.0001
SA_RS12985	carboxymuconolactone decarboxylase family protein	-1.184	<0.0001
SA_RS01110	PTS transporter subunit EIIC	-1.159	<0.0001
SA_RS11550	MAP domain-containing protein	-1.141	<0.0001
hutG	formimidoylglutamase	-1.122	<0.0001
SA_RS02175	DUF1304 domain-containing protein	-1.112	<0.001
hutI	imidazolonepropionase	-1.098	<0.001
esxA	WXG100 family type VII secretion effector EsxA	-1.053	<0.001
SA_RS01115	MurR/RpiR family transcriptional regulator	-1.050	<0.0001
ilvC	ketol-acid reductoisomerase	-1.015	<0.01
crtM	phytoene/squalene synthase family protein	-1.012	<0.01

dhaM	dihydroxyacetone kinase phosphoryl donor subunit DhaM	-1.001	<0.01
SA_RS05995	hypothetical protein	2.336	<0.001
SA_RS05785	hypothetical protein	2.284	<0.01
SA_RS09815	hypothetical protein	1.783	<0.05
SA_RS01490	GntR family transcriptional regulator	1.737	<0.0001
gatC	Asp-tRNA(Asn)/Glu-tRNA(Gln) amidotransferase subunit GatC	1.695	<0.0001
mecA	PBP2a family beta-lactam-resistant peptidoglycan transpeptidase MecA	1.691	<0.0001
malR	LacI family DNA-binding transcriptional regulator	1.638	<0.0001
SA_RS03625	hypothetical protein	1.543	<0.0001
ndk	nucleoside-diphosphate kinase	1.465	<0.0001
SA_RS09010	hypothetical protein	1.419	<0.001
blaZ	penicillin-hydrolyzing class A beta-lactamase BlaZ	1.411	<0.0001
int	tyrosine-type recombinase/integrase	1.368	<0.0001
dltD	D-alanyl-lipoteichoic acid biosynthesis protein DltD	1.352	<0.0001
SA_RS08635	class I SAM-dependent methyltransferase	1.332	<0.0001
SA_RS09105	hypothetical protein	1.331	<0.01
ugpC	sn-glycerol-3-phosphate ABC transporter ATP-binding protein UgpC	1.318	<0.001
dltC	D-alanine—poly (phosphoribitol) ligase subunit 2	1.299	<0.0001
ispE	4-(cytidine 5'-diphospho)-2-C-methyl-D-erythritol kinase	1.237	<0.0001
SA_RS07240	class I SAM-dependent RNA methyltransferase	1.153	<0.0001
ytkD	nucleoside triphosphatase YtkD	1.138	<0.05
dltA	D-alanine--poly(phosphoribitol) ligase subunit DltA	1.133	<0.0001
SA_RS06600	hypothetical protein	1.117	<0.0001
purC	Phosphoribosylaminoimidazolesuccinocarboxamide synthase	1.115	<0.001
SA_RS12160	MurR/RpiR family transcriptional regulator	1.072	<0.001
kdpE *(SCCmec)*	response regulator transcription factor	1.071	<0.01
SA_RS00780	NAD-dependent epimerase/dehydratase family protein	1.066	<0.05
SA_RS10525	helix-turn-helix transcriptional regulator	1.0621	<0.0001
SA_RS09690	ABC transporter ATP-binding protein	1.056	<0.0001
gntR	GntR family transcriptional regulator	1.044	<0.0001
dltB	PG:teichoic acid D-alanyltransferase DltB	1.032	<0.0001
purK	5-(carboxyamino) imidazole ribonucleotide synthase	1.030	<0.0001
sdrC	MSCRAMM family adhesin SdrC	1.025	<0.05
SA_RS05080	isochorismate synthase MenF	1.020	<0.0001
SA_RS09410	helix-turn-helix transcriptional regulator	1.015	<0.0001
SA_RS05495	YlbG family protein	1.005	<0.01

Table S2. MICs of MEM combined with different D-amino acids against MRSA strains

Strains	MEM MIC (μg/mL) at 20 mM D-amino acid of			
	NA	D-Ser	D-Thr	D-Pro
MRSA N315	16	1	16	16
MRSA ATCC 700699	32	4	32	32
MRSA ATCC 43300	8	0.5	4	8
MRSA ATCC 33591	32	0.5	16	32

Table S3. Results of *dlt* operon nucleotide and protein sequence BLAST between *S. aureus* and *E. faecalis*.

Gene name	Nucleotide BLAST			Protein BLAST		
	Query cover	E-value	percent identify	Query cover	E- value	percent identify
dlt operon	33%	2e-32	69.19%	NA	NA	NA
dltA	45%	9e-14	64.72%	100%	5e-132	42.01%
dltB	41%	2e-33	69.19%	99%	6e-107	45.10%
dltC	95%	2e-16	67.54%	100%	3e-25	45.57%
dltD	NA*	NA	NA	95%	8e-58	30.00%

*NA: Not applicable, as *dltD* nucleotide BLAST demonstrated no significant similarity, and Protein BLAST of the whole operon was not conducted considering the intergenic non-coding regions.

Table S4. The primers, plasmids, and bacterial strains used in this study

Strain or Primer	Relevant description or Oligonucleotide (5′-3′)	Source or Application
S. aureus strains		
MRSA N315	Methicillin-resistant *S. aureus* strain	Wildtype strain
E. coli DH5α	Host for plasmid transformation	Vazyme
Plasmid		
pCold I	Cold shock protein expression plasmid, Amp ^{r}	ZOMANBIO
pCold I-N315dltA	pCold I containing MRSA N315 *dltA*	This study
pCold I-N315dltC	pCold I containing MRSA N315 *dlt*C	This study
pCold I-29212dltA	pCold I containing *E. faecalis* 29212 *dltA*	This study
dltA-F	TTAGTGAGCCGTTTCCCAAG	qPCR quantitation of *dltA* expression
dltA-R	CAACGCCAACAGGTAATGTC	
dltB-F	TGGCATGGTATCGAAGTG	qPCR quantitation of *dltB* expression
dltB-R	AACGTGGCGGATGTTTCT	
dltC-F	CAGACGTAGAAATTTTTGAAGAAGG	qPCR quantitation of *dltC* expression
dltC-R	GGTGTTGCCCACTCATCTC	
dltD-F	CGCAGTTGAACTTGCATCAC	qPCR quantitation of *dltD* expression
dltD-R	TTTGAGTTTGAGACATACGAGCA	
rpoB-F	CGTGAAGAAGGCGACGATAC	qPCR quantitation of *rpoB* (internal control) expression
rpoB-R	GTTACCATGTCGACCACACATC	
pCold I-F	ACGCCATATCGCCGAAAGG	For checking of pCold I plasmid
pCold I-R	GGCAGGGATCTTAGATTCTG	
N315DltA-F	CCGCTCGAGATGACAGATATTATTAACAA GCTGC	For amplification of N315 *dltA*
N315DltA-R	CCCAAGCTTTCATCCGTTAATTACCTCTGC AATTT	
N315DltC-F	GGGAATTCCATATGGAATTTAGAGAACAA GTATTAA	For amplification of N315 *dltC*
N315DltC-R	GCTCTAGATCATCGTAACTCTTCTAATGC	
29212DltA-F	GGGAATTCCATATGGAAAAAGTAATTAAT ATGATTCAAACG	For amplification of 29212 *dltA*
29212DltA-R	CCGCTCGAGTCATGTGGCATTCACCTC	

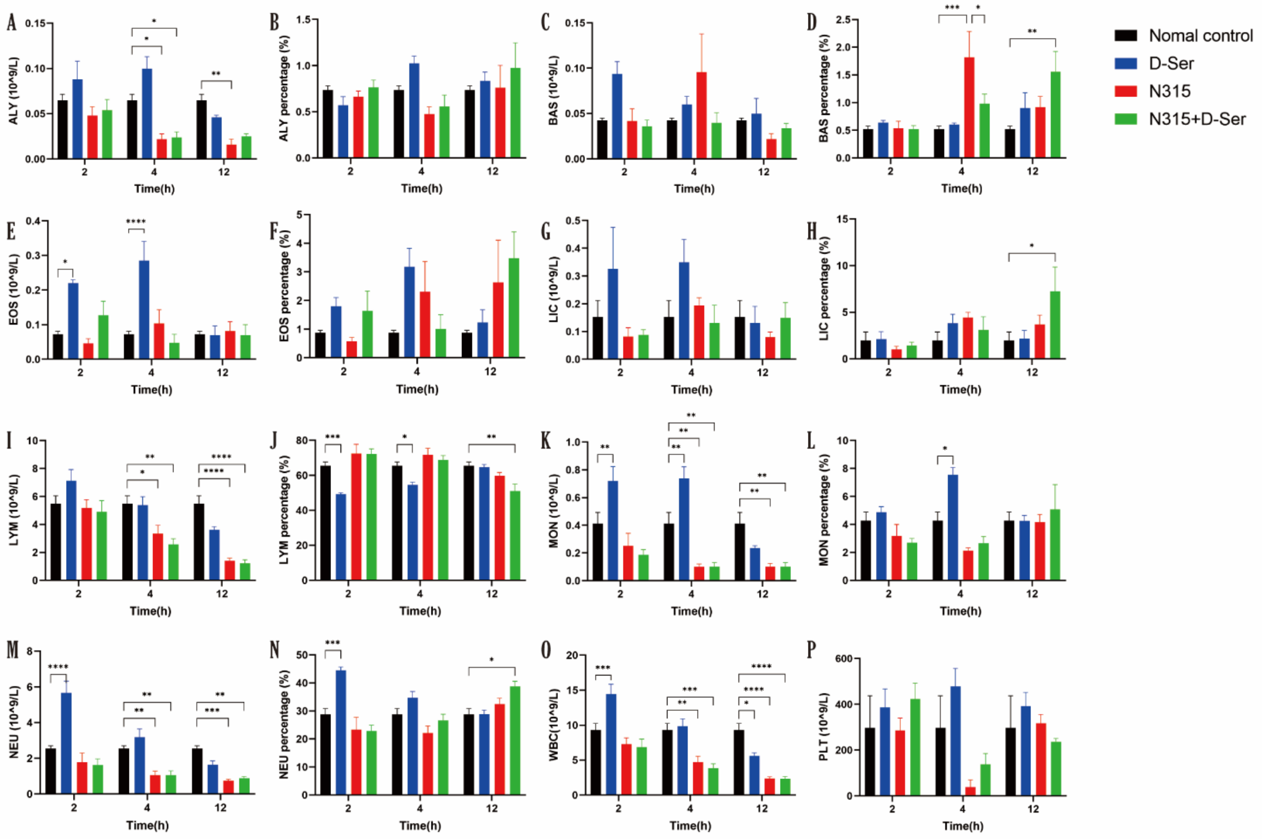

Figure S1. Effects of D-Ser on hematological indices of normal or MRSA N315-infected mice. A-P. The count or percentage of ALY, BAS, EOS, LIC, LYM, MON, NEU, WBC, and PLT in the mouse blood. Data are expressed as mean ± SEM (n = 3~5, the hematological data for some animals were undetectable due to unknown reasons). Differences between groups were analyzed using Two-way ANOVA. * $P < 0.05$, ** $P < 0.01$, *** $P < 0.001$, **** $P < 0.0001$.

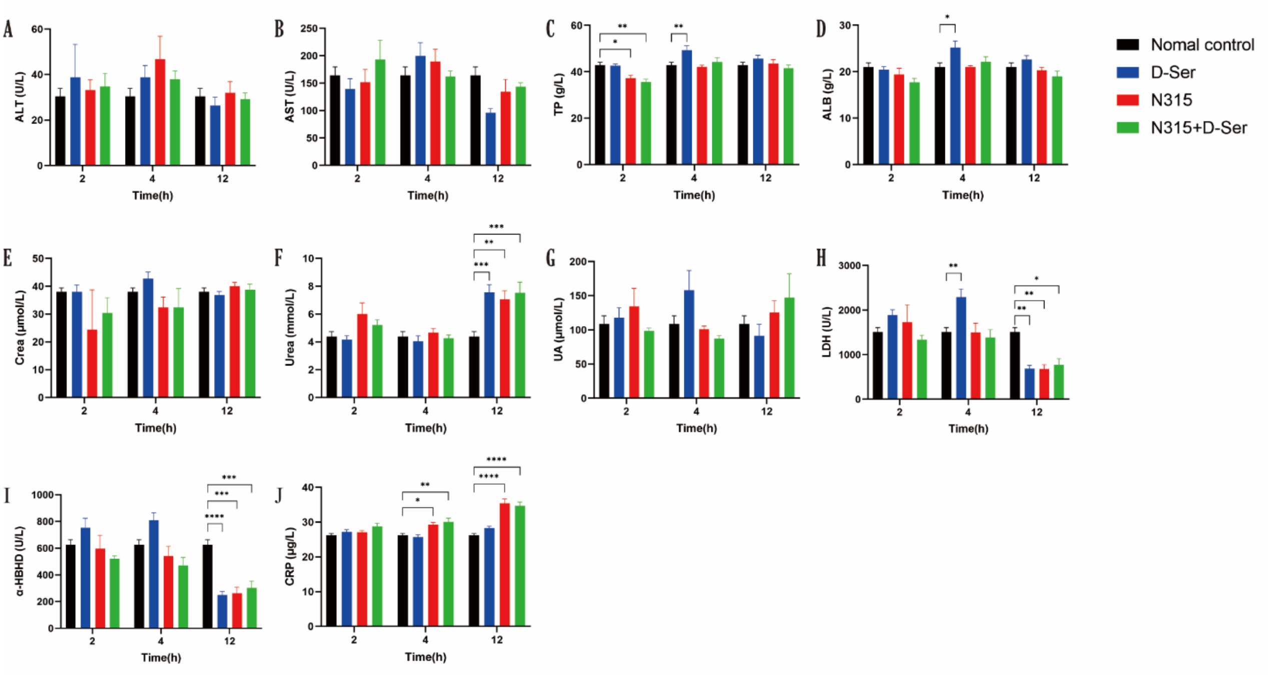

Figure S2. Effects of D-Ser on the serum biochemical indices of normal or MRSA N315-infected mice. A-J. The ALT, AST, TP, ALB, Crea, Urea, UA, LDH, α-HBHD, and CRP levels in the serum. Data are expressed as mean ± SEM (n = 3~5, the biochemical data for some animals were undetectable due to unknown reasons). Differences between groups were analyzed using Two-way ANOVA. * $P < 0.05$, ** $P < 0.01$, *** $P < 0.001$, **** $P < 0.0001$.
